# HC-030031, a TRPA1 selective antagonist, attenuates inflammatory- and neuropathy-induced mechanical hypersensitivity

**DOI:** 10.1186/1744-8069-4-48

**Published:** 2008-10-27

**Authors:** Samer R Eid, Eric D Crown, Eric L Moore, Hongyu A Liang, Kar-Chan Choong, Shelley Dima, Darrell A Henze, Stefanie A Kane, Mark O Urban

**Affiliations:** 1 Department of Pain Research, Neuroscience Drug Discovery, Merck Research Laboratories, West Point, Philadelphia, USA

## Abstract

**Background:**

Safe and effective treatment for chronic inflammatory and neuropathic pain remains a key unmet medical need for many patients. The recent discovery and description of the transient receptor potential family of receptors including TRPV1 and TRPA1 has provided a number of potential new therapeutic targets for treating chronic pain. Recent reports have suggested that TRPA1 may play an important role in acute formalin and CFA induced pain. The current study was designed to further explore the therapeutic potential of pharmacological TRPA1 antagonism to treat inflammatory and neuropathic pain.

**Results:**

The *in vitro *potencies of HC-030031 versus cinnamaldehyde or allyl isothiocyanate (AITC or Mustard oil)-induced TRPA1 activation were 4.9 ± 0.1 and 7.5 ± 0.2 μM respectively (IC_50_). These findings were similar to the previously reported IC_50 _of 6.2 μM against AITC activation of TRPA1 [1]. In the rat, oral administration of HC-030031 reduced AITC-induced nocifensive behaviors at a dose of 100 mg/kg. Moreover, oral HC-030031 (100 mg/kg) significantly reversed mechanical hypersensitivity in the more chronic models of Complete Freunds Adjuvant (CFA)-induced inflammatory pain and the spinal nerve ligation model of neuropathic pain.

**Conclusion:**

Using oral administration of the selective TRPA1 antagonist HC-030031, our results demonstrated that TRPA1 plays an important role in the mechanisms responsible for mechanical hypersensitivity observed in inflammatory and neuropathic pain models. These findings suggested that TRPA1 antagonism may be a suitable new approach for the development of a potent and selective therapeutic agent to treat both inflammatory and neuropathic pain.

## Background

Transient receptor potential ankyrin subfamily, member 1 (TRPA1) is a non-selective cation channel and is the sole mammalian member that defines the TRPA subfamily. TRPA1 was shown to be highly expressed in dorsal root, trigeminal, and nodose ganglia in a specific subpopulation of neurons coexpressing another transient receptor potential family member, TRPV1, and in the hair cells of the inner ear [2-4]. After originally being characterized as a noxious cold-activated ion channel, several reports showed that TRPA1 can be activated by a large number of pungent or irritant compounds, such as cinnamaldehyde, AITC, acrolein, allicin, and formalin, all of which can induce acute pain, hyperalgesia, or neurogenic inflammation in animals and humans [1,5-12]. Additionally, these compounds have been shown to activate primary afferent nociceptors and enhance spontaneous and stimulus-evoked responses of spinal dorsal horn sensory neurons following peripheral application [8,13-15]. More recently, the alpha, beta-unsaturated aldehyde, 4-hydroxy-2-nonenal (HNE) and the electrophilic carbon-containing PGJ2 metabolite, 15dPGJ2, released in response to tissue injury, inflammation, and oxidative stress, were reported to be the first endogenous activators of TRPA1 [10,16,17]. Controversy around the apparent promiscuous activation of TRPA1 by structurally unrelated compounds was diminished by the finding that the majority of TRPA1 activators gate the channel through chemical reactivity of their electrophilic groups with some of the nucleophilic cysteine residues at the N-terminus of the channel [18,19]. In addition, it was shown that TRPA1 can be gated through another mode of activation involving its N-terminal EF-hand calcium binding domain. Two studies suggested that calcium may directly activate the channel by binding to the EF-hand domain, a prerequisite for icilin activation of TRPA1 [20,21].

The above findings that certain pungent and irritant compounds activate TRPA1 led to the suggestion that TRPA1 may be involved in the normal detection of pain. Consistent with such a role, two independent knockout studies showed that TRPA1-mutant mice do not develop acute pain or thermal or mechanical hypersensitivity after intraplantar injection of bradykinin or AITC [6,22]. In one of the two studies, TRPA1-mutant mice also showed reduced sensitivity to intense cold stimulation and a higher threshold of activation in response to painful punctuate mechanical stimulation [22]. In animal models of inflammatory and neuropathic pain, TRPA1 transcripts were up-regulated in dorsal root ganglia. Furthermore, antisense knockdown of TRPA1 suppressed cold hypersensitivity in the SNL (spinal nerve ligation) model and CFA-model of inflammatory pain in rats [23,24]. Finally, TRPA1 appears to be sensitized by NGF and Proteinase-Activated Receptor-2 (PAR2), both of which are known to play a role in inflammatory pain [3,25].

In addition to a potential role in detecting noxious chemical stimuli, there is increasing evidence to suggest that TRP channels have important roles in mechanoreception. Vertebrate TRPA1 was proposed as a candidate mechanically-activated channel in hair cells [2]. Mice lacking TRPA1 function exhibited no hearing deficits although one study showed a lower sensitivity to cutaneous mechanical stimulation [22]. Consistent with a possible role in mechanotransduction, C. *elegans *TRPA1 was shown to be activated by pressure in a heterologous system and to play a key role in mediating mechanosensory functions of this worm [26]. Very recently, Petrus et al., (2007) showed that pharmacological inhibition of TRPA1 using intraplantar injection of the selective antagonist, AP-18, significantly attenuated CFA-induced mechanical hypersensitivity in TRPA1^+/+ ^but not TRPA1^-/- ^mice [27]. In addition, intraperitoneal (IP) administration of another selective TRPA1 antagonist, HC-030031, significantly reduced flinching in both phases of the formalin response *in vivo *[1]. The availability of these reagents provides the opportunity to better understand the role of this channel in pain perception.

Given the oral bioavailability of HC-030031, the present study was undertaken to characterize the antinociceptive effects of this compound in models of inflammatory and neuropathic pain through oral administration. We show that this compound selectively inhibited human TRPA1 activation by cinnamaldehyde with an IC_50 _of 4.9 μM, with no significant off-target activity as assessed against a panel of 48 enzymes, receptors, and transporters known to be involved in pain signalling. Additionally, we report that oral dosing of HC-030031 effectively attenuated mechanical hypersensitivity in models of inflammatory and neuropathic pain, with no observed effects on acute heat sensitivity or motor coordination.

## Results

### HC-030031 selectively inhibits TRPA1 activation by cinnamaldehyde and AITC *in vitro*

The ability of HC-030031 to block TRPA1 activation was tested in a FLIPR calcium-influx assay using HEK-293 cells stably expressing human TRPA1. We first tested the TRPA1 agonists cinnamaldehyde and AITC which evoked dose-dependent calcium influx with EC_50 _values of 15.9 μM and 4.3 μM, respectively (data not shown). Concentrations of HC-030031 from 0.3 to 60 μM were incubated with cells for 10 minutes prior to addition of an EC_60 _concentration of either cinnamaldehyde or AITC. HC-030031 dose-dependently blocked cinnamaldehyde- and AITC-induced calcium influx with IC_50 _values of 4.9 and 7.5 μM, respectively (data not shown). These results are consistent with previous reports [1]. The selectivity of HC-030031 for TRPA1 was evaluated by testing against 48 different enzymes, receptors, and transporters (MDS Pharma Service, Taipei, Taiwan) that have been reported to modulate pain signalling. HC-030031 had no significant activity in any assay at concentrations up to 10 μM (Additional file 1). Previous reports demonstrated that HC-030031 does not block TRPV1, TRPV3 or TRPV4 activation by capsaicin, 2-APB, and 4α-PDD, respectively [1]. In this study, we expanded these findings and assessed the effect of HC-030031 on TRPM8. We found that HC-030031 did not block TRPM8 activation at concentrations up to 30 μM (data not shown).

### Allyl Isothiocyanate-Induced Nocifensive Behaviors are Attenuated by HC-030031

**Figure 1:**
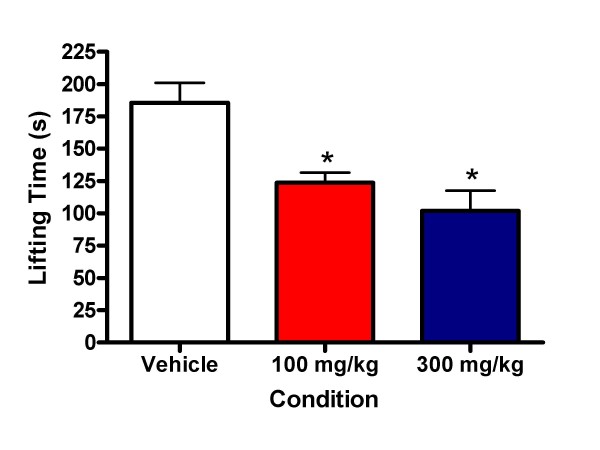
**The effect of TRPA1 antagonism on *AITC*-induced nocifensive behaviors**. Both 100 mg/kg (red bar) and 300 mg/kg (blue bar) of HC-030031 significantly reduced lifting behavior relative to vehicle treated rats given plantar injection of 1% AITC (white bar; * p, 0.05).

The ability of HC-030031 to block the direct activation of TRPA1 was investigated by examining the potential inhibition of AITC-induced spontaneous hindpaw lifting. AITC induced significant spontaneous lifting behavior during the 5 minute observation period following intraplantar injection. The mean lifting duration for the vehicle group (± SEM) was 182.32 s (± 14.97). One hour post-oral administration (Tmax = 45 minutes after oral administration in rats; US patent application 2007/0219222 A1), HC-030031 significantly reduced the lifting duration following 1% AITC injection (ANOVA, p < 0.001, Figure 1). The 100 mg/kg group had an average duration of 123.66 (± 7.44) s and the 300 mg/kg group had an average duration of 107.52 (± 14.54) s following AITC injection. Post hoc tests indicated that rats given either 100 or 300 mg/kg HC-030031 had significantly shorter lifting durations than vehicle treated rats (p < 0.05). These findings are consistent with the results of McNamara and colleagues (2007). The average plasma exposure of HC-030031 in this study was consistent with the levels found in the CFA and SNL studies; 5.10 (± 0.67) μM for the 100 mg/kg group and 9.74 (± 0.37) μM for the 300 mg/kg group.

### CFA Induced Mechanical Hypersensitivity is Attenuated by HC-030031

**Figure 2:**
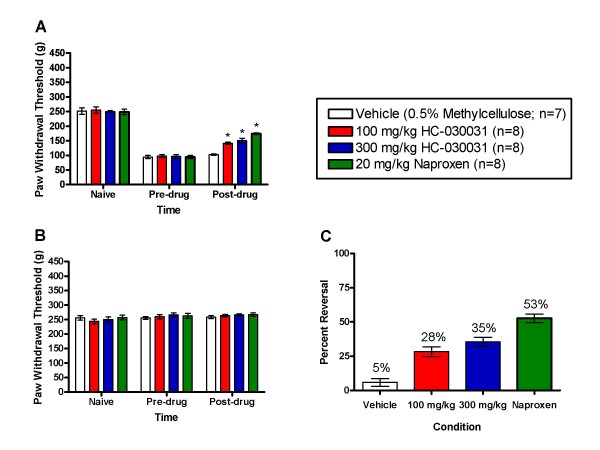
**The effect of TRPA1 antagonism on complete Freund's adjuvant induced mechanical hypersensitivity**. A. Paw withdrawal thresholds for the injected hind paw were assessed using the Randall-Selitto device prior to intraplantar CFA injections, prior to dosing with vehicle (white bars), 20 mg/kg naproxen (green bars), 100 mg/kg HC-030031 (red bars) or 300 mg/kg HC-030031 (blue bars), and at one hour post *p.o. *dosing. Naproxen, 100 mg/kg and 300 mg/kg HC-030031 produced a significant attenuation in mechanical hypersensitivity at one hour post dose (*p < 0.05). B. Paw withdrawal thresholds for the noninjected hind paw assessed using the Randall-Selitto device prior to intraplantar CFA injections, prior to dosing with vehicle (white bars), 20 mg/kg naproxen (green bars), 100 mg/kg HC-030031 (red bars) or 300 mg/kg HC-030031 (blue bars), and at one hour post *p.o. *dosing. C. Percent reversal seen at one hour post *p.o. *dosing with vehicle (white bars), 20 mg/kg naproxen (green bars), 100 mg/kg HC-030031 (red bars) or 300 mg/kg HC-030031 (blue bars).

To examine the role of TRPA1 antagonism in inflammatory pain, we tested HC-030031 using a common measure of mechanical hypersensitivity, the Randall-Selitto test. Injection of CFA into the left hindpaw of the rat produced mechanical hypersensitivity in all subjects at the 24 hour time point, as evidenced by a significant decrease in mechanical threshold. Compared to vehicle controls, both the positive comparator, naproxen, and the TRPA1 antagonist, HC-030031, significantly attenuated the mechanical hypersensitivity at 1 hr post dosing (RMANOVA, p < 0.001; Figure 2 panel A). Post hoc tests found that the naproxen (20 mg/kg) and both 100 and 300 mg/kg HC-030031 groups had significantly higher mechanical thresholds than the vehicle controls (p < 0.05). As can be seen in Figure 2 (panel B), neither dose of HC-030031 had an impact on mechanical thresholds for the uninjected paw. Panel C of Figure 2 demonstrates the percent reversal [calculated as (post-drug - pre-drug)/(naïve-pre-drug)*100] for vehicle (5%), naproxen (53%), and both doses of HC-030031 (28 and 35% for 100 and 300 mg/kg, respectively). In this study, the average plasma exposure (± SEM) of HC-030031 measured immediately after testing was 8.23 (± 0.55) μM for the 100 mg/kg group and 16.33 (± 1.86) μM for the 300 mg/kg group.

### Spinal Nerve Ligation Induced Mechanical Hypersensitivity is Attenuated by HC-030031

**Figure 3:**
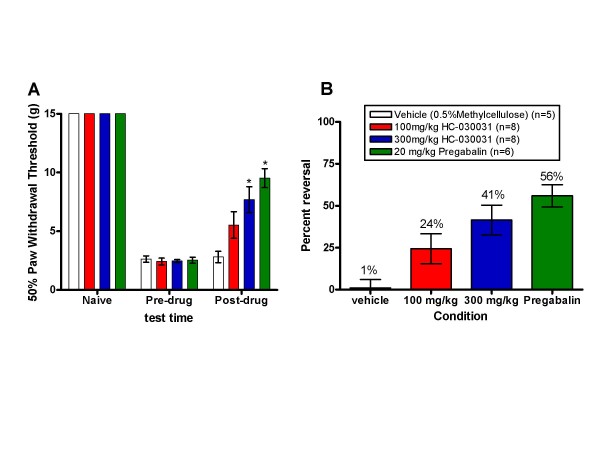
**The effect of HC-030031 on spinal nerve ligation induced mechanical hypersensitivity**. A. Fifty percent paw withdrawal thresholds for the L5/L6 spinal nerve ligated rats assessed using the Dixon up/down method prior to surgery, prior to dosing with vehicle (white bars), 20 mg/kg pregabalin (green bars), 100 mg/kg HC-030031 (red bars) or 300 mg/kg HC-030031 (blue bars), and at one hour post p.o. dosing. Pregabalin and 300 mg/kg HC-030031 produced a significant attenuation in mechanical hypersensitivity at one hour post dose (*p < 0.05). B. Percent reversal seen at one hour post p.o. dosing with vehicle (white bars), 20 mg/kg pregabalin (green bars), 100 mg/kg HC-030031 (red bars) or 300 mg/kg HC-030031 (blue bars).

Next, a possible role for TRPA1 in spinal nerve ligation induced neuropathic pain was investigated using HC-030031. By 6 weeks post surgery, rats with L5/L6 ligation displayed significant mechanical hypersensitivity. This hypersensitivity was significantly reversed by HC-030031 (24 and 41% for 100 and 300 mg/kg, respectively) and pregabalin (56%; RMANOVA, p < 0.003, Figure 3, panel A). Post hoc tests indicated that rats given the 300 mg/kg dose of HC-030031 or pregabalin (20 mg/kg) had significantly higher mechanical thresholds than vehicle controls (p < 0.05). Panel B of Figure 3 demonstrates the percent reversal for vehicle, pregabalin, 100 mg/kg and 300 mg/kg HC-030031. The average plasma exposure (± SEM) of HC-030031 measured immediately after testing was 6.09 (± 1.25) μM for the 100 mg/kg group and 7.55 (± 0.91) μM for the 300 mg/kg group.

### HC-030031 Does Not Alter Locomotor Coordination or Thermal Sensitivity

**Figure 4:**
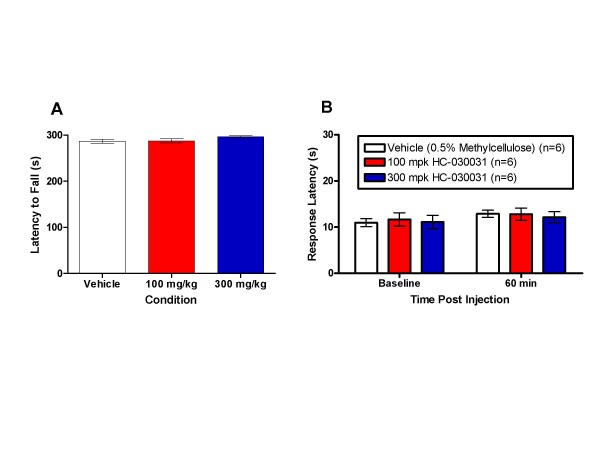
**The effect of HC-030031 on locomotor coordination and thermal sensitivity**. A. Neither the 100 mg/kg (red bar) nor the 300 mg/kg (blue bar) dose of HC-030031 had a significant impact on the latency to fall off the rotarod during locomotor testing relative to vehicle treated rats (white bar). B. HC-030031 also failed to produce a significant change in thermal latencies on the hot plate test for rats dosed at 100 mg/kg (red bar) or 300 mg/kg (blue bar) relative to vehicle treated rats (white bar).

To discount the possibility that TRPA1 antagonism produced locomotor effects that biased the current results, rats were dosed with 100 or 300 mg/kg and tested using the accelerating rotarod test at 1 hr post dose. An ANOVA found no significant differences between the vehicle and drug groups (p > 0.05; Figure 4, panel A). This finding is consistent with other reports for HC-030031 [1]. Lastly, HC-030031 was tested for its effect on thermal reactivity using the hot plate test. A RMANOVA performed on the data found no significant differences between the groups (p > 0.05), suggesting that TRPA1 antagonism does not alter basal thermal latencies (Figure 4, panel B). The average plasma exposure (± SEM) for the rotarod experiment was 4.31 (± 0.36) μM for the 100 mg/kg group and 10.93 (± 0.44) μM for the 300 mg/kg group. For the hot plate test, the average plasma exposure (± SEM) was 4.99 (± 0.69) μM for the 100 mg/kg group and 7.21 (± 0.58) μM for the 300 mg/kg group.

## Discussion

In the present study, an orally bioavailable TRPA1 receptor antagonist, HC-030031, was used to further evaluate the role of TRPA1 receptors in models of inflammatory and neuropathic pain, and in tests of acute heat sensitivity and motor coordination. HC-030031 is a selective small molecule TRPA1 receptor antagonist (human IC_50 _= 4.9 μM). This compound previously was shown to inhibit AITC and formalin-evoked TRPA1 currents *in vitro*, and inhibit AITC and formalin-induced nociceptive behaviors *in vivo *following intraperitoneal dosing[1]. Moreover, HC-030031 did not display any significant binding to 41 other receptors, ion channels, and transporters nor functional modulation of 7 enzymes that are known to modulate pain signaling in a target screen (< 40% effect at 10 μM). To confirm *in vivo *TRPA1 receptor engagement by HC-030031, effects of this compound on AITC-induced nociceptive behaviors were determined. When administered prior to application of AITC, HC-030031 (100 and 300 mg/kg, p.o.) was found to significantly inhibit AITC-induced nociceptive behaviors, suggesting that these doses effectively block TRPA1 receptor activation. Moreover, the fact that plasma exposures of 5.1 and 9.7 μM were obtained following oral dosing of 100 and 300 mg/kg, respectively, supports good oral bioavailability of this compound in rats.

To examine the role of TRPA1 receptors in inflammatory pain states, the effect of HC-030031 was evaluated in the CFA model of inflammatory pain. CFA is a commonly used rodent model of subchronic inflammatory pain [28], and in the present study oral administration of HC-030031 was found to reverse CFA-induced mechanical hypersensitivity, although efficacy was somewhat less than that of the positive comparator naproxen. These results are consistent with a number of previous reports that have supported a role for TRPA1 receptors in inflammatory pain. Using a small molecule TRPA1 receptor antagonist designated AP18 (mouse IC_50 _= 4.5 μM), Petrus et al. (2007) found that intra-paw injection of this compound completely reversed CFA-induced mechanical hypersensitivity in wild-type, but not TRPA1 knock-out mice. Additionally, this compound was found to reverse partially CFA-induced cold hypersensitivity. In a separate study using TRPA1 antisense oligodeoxynucleotides, Obata et al. (2005) found that intrathecal TRPA1 antisense administration inhibited CFA-induced cold allodynia, but not heat or mechanical hypersensitivity. Although it is unclear why effects on mechanical hypersensitivity were not observed, differences in experimental design may account for the differing results. For example, Obata et al. (2005) reported that antisense treatment only reduced the increased TRPA1 receptor expression following CFA injection, and thus greater receptor blockade/inactivation may be required to inhibit mechanical hypersensitivity. Additionally, differences in the mechanical stimuli used in these studies, and time of treatment relative to CFA injection (i.e. development vs maintenance) may account for the differing results. The contribution of TRPA1 receptors to inflammatory pain has been suggested to occur as a consequence of receptor sensitization and/or activation by a variety of proinflammatory mediators including bradykinin [5,6,22], protease activated receptor 2 (PAR2) agonists[25], and prostaglandins [16,29,30]. The variety of inflammatory mediators that have been found to directly activate or indirectly sensitize TRPA1 is consistent with the results from the present study supporting a role for TRPA1 in inflammatory pain.

In addition to effects on inflammatory pain, we found that oral administration of HC-030031 significantly reversed tactile hypersensitivity in the SNL model of neuropathic pain. The efficacy observed at the highest dose tested was comparable to the positive comparator pregabalin, which is considered by many to be the first-line treatment for neuropathic pain [31] (for recent review see Gajraj 2007). TRPA1 has been previously implicated in neuropathic pain based on the observation that TRPA1 mRNA expression was found to be increased in uninjured small diameter TrkA^+ ^neurons in the L4 DRG following L5 SNL, and the time course of this upregulation was correlated with the development of cold hypersensitivity [23]. More direct evidence for a role of TRPA1 in the maintenance of cold hypersensitivity in this study was provided by the observation that acute knock-down of TRPA1 following intrathecal administration of antisense oligodeoxynucleotides inhibited cold hypersensitivity. Although these data suggest an important role for TRPA1 in the maintenance of pain following neuropathy, the endogenous mediators and/or mechanisms responsible for TRPA1 activation in this case are less clear. In contrast to the efficacy achieved in the SNL model with pharmacological blockade of TRPA1, both mice lacking the function of TRPA1 and their wildtype littermates developed significant mechanical hypersensitivity in the spared nerve injury (SNI) model. The reason for this discrepancy is unclear, but one possibility is the upregulation of a protein that could compensate for the function of TRPA1. Another possibility is the potential embryonic rewiring of the neuronal circuitries expressing TRPA1 possibly masking the phenotype. Finally, while both SNL and SNI models of neuropathic pain are characterized by heightened sensitivity to mechanical stimulation, they differ in the nature of the injury.

In the present study, the plasma concentrations (i.e. ~5.0–10 μM) that produced antinociceptive efficacy were in a similar range compared to the *in vitro *potency values on TRPA1. This finding was somewhat unusual, since plasma exposures required to produce antinociceptive efficacy are typically significantly greater than the *in vitro *potency values for analgesic targets. Nevertheless, the fact that HC-030031 did not display off-target activity in a target screen, and was found to inhibit AITC-induced nociceptive behaviors at antinociceptive doses, suggests that the mechanism of action likely involves TRPA1 blockade, although other mechanisms cannot be ruled out.

While HC-030031 was effective in reversing mechanical hypersensitivity in models of inflammatory and neuropathic pain, it remains unclear how TRPA1 activation contributes to mechanosensation. Invertebrate and mammalian TRPA1 receptors were shown to be involved in mechanoreception [2,26]. However, no data supporting a direct activation of this channel by mechanical stimulation is present to date. One possibility is that in response to mechanical stimulation, damaged/inflamed tissues release endogenous reactive mediators or respond by an increase in intracellular calcium, both known to directly activate TRPA1. Alternatively, inflammatory mediators may sensitize and/or lower TRPA1 threshold to mechanical stimulation leading to the development of mechanical hypersensitivity. Following injury, TRPA1 might enhance the mechanoresponsiveness of other proteins through direct or indirect mechanisms.

The observed efficacy of HC-030031 in inflammatory and neuropathic pain appears to not involve any coincident changes in acute heat sensitivity or motor coordination. Oral administration of HC-030031 failed to have any effect on hot plate response latencies or rotarod performance at doses which produced efficacy in the inflammatory and neuropathic pain models. Plasma exposures from these experiments were comparable to exposures obtained in the persistent pain model experiments, confirming lack of effect at doses that produced efficacy against inflammatory and neuropathic pain. The ineffectiveness of HC-030031 in the hot plate test is consistent with previous data showing a lack of noxious heat phenotype in TRPA1 knock-out mice and supports the notion that TRPA1 is not a heat sensor [6,22]. Additionally, the results from the hot plate experiment are consistent with the lack of effect of HC-030031 on contralateral, uninjured hind paw withdrawal thresholds, in response to mechanical stimulation, in the CFA model suggesting that the antinociceptive effects are specific to injury-induced hypersensitivity. That HC-030031 had no effect on motor coordination at antinociceptive doses is consistent with a previous report demonstrating that the efficacy of this compound is likely specific to effects on sensory transmission and is not related to motor dysfunction [1].

## Conclusion

In summary, the data from the present study have demonstrated for the first time that an orally administered small molecule TRPA1 receptor antagonist can effectively inhibit mechanical hypersensitivity in rodent models of inflammatory and neuropathic pain at doses that do not affect acute nociception or motor coordination. These results further suggest that TRPA1 plays an important role in nociceptive transmission and support the development of potential novel analgesics directed against this target.

## Methods

### Cell Line Generation

Full length human TRPA1 cDNA was purchased from Origene Technologies (Rockville, MD) and subcloned into pcDNA5/FRT/V5-His TOPO TA vector (Invitrogen, Carlsbad, CA). Sequence analysis identified two point mutations within the coding region of TRPA1 which were corrected using the QuickChange II site-directed mutagenesis kit (Stratagene, La Jolla, CA). A HEK-293 cell-line stably expressing TRPA1 was generated by selection in 100 μg/mL hygromycin B using the Invitrogen Flp-In system.

### FLIPR Assay

HEK-293 cells stably expressing human TRPA1 were plated into 384-well plates at a density of 20,000 cells/well 24 hours prior to assaying. On the day of assay, cells were loaded with 4 μM Fluo-4 dye (Invitrogen) and 0.08% pluronic acid (Invitrogen) for 1 hour at room temperature in assay buffer consisting of Hank's balanced salt solution (Invitrogen) supplemented with 20 mM HEPES (Invitrogen), 2.5 mM probenecid (Sigma, St. Louis, MO), and 4% TR-40 (Invitrogen). Calcium influx assays were performed using the Fluorometric Imaging Plate Reader (FLIPR) TETRA (Molecular Devices, Sunnyvale, CA). Concentration-response curves were generated for the TRPA1 agonists cinnamaldehyde and AITC prior to antagonist testing so EC_60 _concentrations could be determined. Titrations of HC-030031 were made from a DMSO stock solution and DMSO was kept to a constant of 0.4% in the assay. The antagonist was incubated with the cells for 10 minutes before the addition of an EC_60 _concentration of either cinnamaldehyde (18 μM) or AITC (6 μM) and calcium influx was monitored for an additional 10 minutes.

### Subjects

Male Sprague-Dawley rats (200–500 g) from Taconic (New York) were used in all experiments. Animals in all experiments were allowed ad lib access to food and water until 16 hrs prior to behavioral testing, at which time the animals were food deprived. All animal procedures were approved by the Merck Institutional Animal Care and Use Committee and were performed in accordance with The Guide for the Care and Use of Laboratory Animals.

### Surgery

To examine the effects of HC-030031 on neuropathic pain, male Sprague-Dawley rats were given a spinal nerve ligation (SNL) injury following the procedure of Kim and Chung (1992) [32]. Briefly, rats were anesthetized with 2–3% isofluorane and the region on the back at the site of surgery was shaved and cleaned with topical Betadine and ethanol. Once an adequate plane of anesthesia was attained (as assessed by the loss of hindlimb reflexes), a dorsal midline incision was made from approximately spinal nerve L3 to S2. A combination of sharp and blunt dissection probes were used to expose the L6/S1 posterior interarticular process. The L6 transverse process was visualized and removed and the L4 and L5 spinal nerves were exposed distal to their emergence from the intervertebral foramina. The L5 and L6 nerves were tightly ligated with 6-0 silk suture. Following ligation, the muscle and skin were closed with 4-0 absorbable sutures.

### Compounds

HC-030031 (100, 300 mg/kg) was synthesized at Chembridge (San Diego, CA). For all experiments, HC-030031 was suspended in 0.5% Methylcellulose and the drug was dosed *p.o*. at a volume of 10 ml/kg. Naproxen (20 mg/kg; Sigma Aldrich) was dissolved in sterile water and dosed *p.o*. to serve as a positive comparator for the CFA experiment. Pregabalin (20 mg/kg) was dissolved in sterile water and dosed *p.o*. to serve as a positive comparator for the neuropathic pain experiment.

### Experimental Procedures

#### Complete Freund's Adjuvant (CFA) Induced Mechanical Hypersensitivity

Prior to injection with CFA, rats were given baseline testing to establish mechanical thresholds using the Randall-Selitto device (Ugo Basile, Switzerland). After baseline thresholds were established, rats were injected with 100 μl of 1 mg/ml CFA into the left hindpaw. Twenty-four hrs later, injected rats were tested for the development of mechanical hypersensitivity. Once mechanical hypersensitivity had been demonstrated, rats were counterbalanced into groups and dosed p.o. with vehicle (0.5% Methylcellulose), 100 mg/kg or 300 mg/kg of HC-030031 or 20 mg/kg naproxen. Mechanical thresholds were then reassessed at 1 hr post dosing. Both hindpaws were tested so that each animal's untreated paw could serve as a control. In addition, testing was done by an experimenter blinded to treatment condition. Immediately after the last test, rats were euthanized with CO_2 _and both brain and plasma samples were taken in order to determine the exposure levels for HC-030031.

#### Spinal Nerve Ligation Induced Neuropathic Pain

Graded von Frey filaments (Stoelting) were applied to the hindpaw to assess 50% withdrawal thresholds using the Dixon up/down method [33] in rats that had received L5/L6 spinal nerve ligation. For the current study, only rats that developed mechanical hypersensitivity (as defined as a 50% mechanical withdrawal threshold of 4 g or less) were examined. At six weeks post SNL surgery, rats were given a baseline test and the groups were counterbalanced prior to being dosed *p.o*. with vehicle (0.5% Methylcellulose), 100 or 300 mg/kg HC-030031, or 20 mg/kg pregabalin. One hour after dosing, rats were tested with graded von Frey filaments to establish 50% withdrawal thresholds. Testing was performed by an experimenter blinded to treatment condition. Immediately after the last test, rats were euthanized with CO_2 _and both brain and plasma samples were taken in order to determine the exposure levels for HC-030031.

#### Allyl Isothiocyanate-Induced Nocifensive Behaviors

The TRPA1 agonist, allyl isothiocyanate (AITC; Fluka) was used to activate peripheral TRPA1 receptors and elicit nocifensive behaviors in the rat. Prior parametric work (data not shown) indicated that 1% AITC was the lowest concentration that produced a maximal behavioral response in this assay. Rats were dosed *p.o. *with vehicle (0.5% Methylcellulose), 100 or 300 mg/kg of HC-030031 one hour prior to testing and allowed to acclimate to Plexiglass observation chambers for 15 minutes prior to AITC injection. AITC (1%) was injected into the plantar surface of the left hindpaw and the duration of time spent lifting the paw was recorded using a stop watch for 5 minutes post injection by an experimenter blinded to treatment condition. Immediately after the 5 min observation period, rats were euthanized with CO_2 _and both brain and plasma samples were taken in order to determine the exposure levels for HC-030031.

#### Rotarod Test of Locomotor Coordination

The effects of HC-030031 on locomotor coordination were assessed using the rotarod (IITC Life Science, Woodland Hills CA). Prior to testing, each rat was given a baseline test to establish that there were no preexisting locomotor deficits. To pass the baseline test, rats were required to stay on the rotarod for 120 seconds at a speed of 12 rpm. After the baseline test, rats were dosed *p.o. *with vehicle (0.5% Methylcellulose), 100 or 300 mg/kg HC-030031. One hour post dosing rats were tested for deficits in locomotor coordination. This test involved accelerating the speed of the rotarod from 4 rpm to 33 rpm over the course of a five minute testing interval. Latency to fall off the rotarod served as the measure. Testing was performed by an experimenter blinded to treatment condition. Immediately after testing, rats were euthanized with CO_2 _and both brain and plasma samples were taken in order to determine the exposure levels for HC-030031.

#### Hot Plate Test

To examine the effects of HC-030031 on heat sensitivity, rats were tested using the hot plate (Ugo Basile, Italy). Rats were placed on the 52 degree C hot plate and the latency to lick the hindpaw was measured. Following initial testing, rats were counterbalanced into groups and dosed *p.o. *with vehicle (0.5% Methylcellulose), 100 or 300 mg/kg HC-030031. One hour post dosing, rats were again assessed on the 52 degree C hot plate to establish changes in heat sensitivity. Testing was performed by an experimenter blinded to treatment condition. Immediately after testing, rats were euthanized with CO_2 _and both brain and plasma samples were taken in order to determine the exposure levels for HC-030031.

### Determination of HC-030031 plasma concentrations

Plasma concentration of HC-030031 was determined using LC-MS/MS with a heated nebulizer interface (Sciex 4000, Sciex, OT, Canada) in the positive ion mode. Chromatography is accomplished with a Phenomenex Luna C18 (50 × 2 mm, 5 μm) column using reverse phase mobile phase under fast gradient conditions. Quantitation was based on selected reaction monitoring of the precursor/product ion pairs for HC-030031 and the internal standard. Concentrations of HC-030031 were determined by interpolation from a standard curve prepared in blank rat plasma.

### Statistics

Repeated measures analysis of variance (RMANOVA) was used to examine changes in reactivity in the CFA-induced mechanical hypersensitivity and hot plate tests. RMANOVA utilizing the Greenhouse-Geisser correction to compensate for unequal error variance was used to analyze the SNL-mechanical sensitivity data and this test was followed up with a post hoc Dunnett's test to distinguish differences between the groups. One way ANOVA was used to analyze the data from the allyl isothiocyanate induced nocifensive assay and the rotarod experiment. Unless otherwise specified, post hoc tests were performed using the Tukey test. In all cases, statistical significance was set at p < 0.05.

## Competing interests

All authors are employees of Merck Research Laboratories and may own stock options for Merck & Co., Inc.

## Authors' contributions

ELM conducted the FLIPR assays and EDC performed dosing, behavioral testing, and tissue collection. Both wrote portions of the methods and results. HAL, KCC, and SD assisted with the dosing, behavioral testing, and tissue collection. SRE and MOU advised and supervised behavioral assays, wrote the background and discussion, and edited the manuscript. SRE conceived, designed and coordinated the study. DAH and SAK discussed and contributed to study design, and edited the manuscript. DAH wrote the abstract. All authors read and approved the final manuscript.

## Supplementary Material

Additional file 1 **Table 1: *In-vitro *pharmacological selectivity of HC-030031**. Radioligand binding or enzymatic assays results are summarized as the percent inhibition of specific binding or enzymatic activity. HC-030031 exhibited no significant activity in all assays employed (significance criteria is ≥ 50% of maximal stimulation or inhibition; MDS Pharma Service, Taipei, Taiwan.). Click here for file

## References

[B1] McNamara CR, Mandel-Brehm J, Bautista DM, Siemens J, Deranian KL, Zhao M, Hayward NJ, Chong JA, Julius D, Moran MM, Fanger CM (2007). TRPA1 mediates formalin-induced pain. Proc Natl Acad Sci USA.

[B2] Corey DP, Garcia-Anoveros J, Holt JR, Kwan KY, Lin SY, Vollrath MA, Amalfitano A, Cheung EL, Derfler BH, Duggan A, Geleoc GS, Gray PA, Hoffman MP, Rehm HL, Tamasauskas D, Zhang DS (2004). TRPA1 is a candidate for the mechanosensitive transduction channel of vertebrate hair cells. Nature.

[B3] Diogenes A, Akopian AN, Hargreaves KM (2007). NGF up-regulates TRPA1: implications for orofacial pain. J Dent Res.

[B4] Story GM, Peier AM, Reeve AJ, Eid SR, Mosbacher J, Hricik TR, Earley TJ, Hergarden AC, Andersson DA, Hwang SW, McIntyre P, Jegla T, Bevan S, Patapoutian A (2003). ANKTM1, a TRP-like channel expressed in nociceptive neurons, is activated by cold temperatures. Cell.

[B5] Bandell M, Story GM, Hwang SW, Viswanath V, Eid SR, Petrus MJ, Earley TJ, Patapoutian A (2004). Noxious cold ion channel TRPA1 is activated by pungent compounds and bradykinin. Neuron.

[B6] Bautista DM, Jordt SE, Nikai T, Tsuruda PR, Read AJ, Poblete J, Yamoah EN, Basbaum AI, Julius D (2006). TRPA1 mediates the inflammatory actions of environmental irritants and proalgesic agents. Cell.

[B7] Bautista DM, Movahed P, Hinman A, Axelsson HE, Sterner O, Hogestatt ED, Julius D, Jordt SE, Zygmunt PM (2005). Pungent products from garlic activate the sensory ion channel TRPA1. Proc Natl Acad Sci USA.

[B8] Jordt SE, Bautista DM, Chuang HH, McKemy DD, Zygmunt PM, Hogestatt ED, Meng ID, Julius D (2004). Mustard oils and cannabinoids excite sensory nerve fibres through the TRP channel ANKTM1. Nature.

[B9] Macpherson LJ, Geierstanger BH, Viswanath V, Bandell M, Eid SR, Hwang S, Patapoutian A (2005). The pungency of garlic: activation of TRPA1 and TRPV1 in response to allicin. Curr Biol.

[B10] Macpherson LJ, Xiao B, Kwan KY, Petrus MJ, Dubin AE, Hwang S, Cravatt B, Corey DP, Patapoutian A (2007). An ion channel essential for sensing chemical damage. J Neurosci.

[B11] Namer B, Seifert F, Handwerker HO, Maihofner C (2005). TRPA1 and TRPM8 activation in humans: effects of cinnamaldehyde and menthol. Neuroreport.

[B12] Ward L, Wright E, McMahon SB (1996). A comparison of the effects of noxious and innocuous counterstimuli on experimentally induced itch and pain. Pain.

[B13] Jancso N, Jancso-Gabor A, Szolcsanyi J (1967). Direct evidence for neurogenic inflammation and its prevention by denervation and by pretreatment with capsaicin. Br J Pharmacol Chemother.

[B14] Merrill AW, Cuellar JM, Judd JH, Carstens MI, Carstens E (2008). Effects of TRPA1 agonists mustard oil and cinnamaldehyde on lumbar spinal wide-dynamic range neuronal responses to innocuous and noxious cutaneous stimuli in rats. J Neurophysiol.

[B15] Puig S, Sorkin LS (1996). Formalin-evoked activity in identified primary afferent fibers: systemic lidocaine suppresses phase-2 activity. Pain.

[B16] Taylor-Clark TE, Undem BJ, Macglashan DW, Ghatta S, Carr MJ, McAlexander MA (2008). Prostaglandin-induced activation of nociceptive neurons via direct interaction with transient receptor potential A1 (TRPA1). Mol Pharmacol.

[B17] Trevisani M, Siemens J, Materazzi S, Bautista DM, Nassini R, Campi B, Imamachi N, Andre E, Patacchini R, Cottrell GS, Gatti R, Basbaum AI, Bunnett NW, Julius D, Geppetti P (2007). 4-Hydroxynonenal, an endogenous aldehyde, causes pain and neurogenic inflammation through activation of the irritant receptor TRPA1. Proc Natl Acad Sci USA.

[B18] Hinman A, Chuang HH, Bautista DM, Julius D (2006). TRP channel activation by reversible covalent modification. Proc Natl Acad Sci USA.

[B19] Macpherson LJ, Dubin AE, Evans MJ, Marr F, Schultz PG, Cravatt BF, Patapoutian A (2007). Noxious compounds activate TRPA1 ion channels through covalent modification of cysteines. Nature.

[B20] Doerner JF, Gisselmann G, Hatt H, Wetzel CH (2007). Transient receptor potential channel A1 is directly gated by calcium ions. J Biol Chem.

[B21] Zurborg S, Yurgionas B, Jira JA, Caspani O, Heppenstall PA (2007). Direct activation of the ion channel TRPA1 by Ca2+. Nat Neurosci.

[B22] Kwan KY, Allchorne AJ, Vollrath MA, Christensen AP, Zhang DS, Woolf CJ, Corey DP (2006). TRPA1 contributes to cold mechanical, chemical nociception but is not essential for hair-cell transduction. Neuron.

[B23] Katsura H, Obata K, Mizushima T, Yamanaka H, Kobayashi K, Dai Y, Fukuoka T, Tokunaga A, Sakagami M, Noguchi K (2006). Antisense knock down of TRPA1, but not TRPM8, alleviates cold hyperalgesia after spinal nerve ligation in rats. Exp Neurol.

[B24] Obata K, Katsura H, Mizushima T, Yamanaka H, Kobayashi K, Dai Y, Fukuoka T, Tokunaga A, Tominaga M, Noguchi K (2005). TRPA1 induced in sensory neurons contributes to cold hyperalgesia after inflammation and nerve injury. J Clin Invest.

[B25] Dai Y, Wang S, Tominaga M, Yamamoto S, Fukuoka T, Higashi T, Kobayashi K, Obata K, Yamanaka H, Noguchi K (2007). Sensitization of TRPA1 by PAR2 contributes to the sensation of inflammatory pain. J Clin Invest.

[B26] Kindt KS, Viswanath V, Macpherson L, Quast K, Hu H, Patapoutian A, Schafer WR (2007). Caenorhabditis elegans TRPA-1 functions in mechanosensation. Nat Neurosci.

[B27] Petrus M, Peier AM, Bandell M, Hwang SW, Huynh T, Olney N, Jegla T, Patapoutian A (2007). A role of TRPA1 in mechanical hyperalgesia is revealed by pharmacological inhibition. Mol Pain.

[B28] Stein C, Millan MJ, Herz A (1988). Unilateral inflammation of the hindpaw in rats as a model of prolonged noxious stimulation: alterations in behavior and nociceptive thresholds. Pharmacol Biochem Behav.

[B29] Burian M, Geisslinger G (2005). COX-dependent mechanisms involved in the antinociceptive action of NSAIDs at central and peripheral sites. Pharmacol Ther.

[B30] Materazzi S, Nassini R, Andre E, Campi B, Amadesi S, Trevisani M, Bunnett NW, Patacchini R, Geppetti P (2008). Cox-dependent fatty acid metabolites cause pain through activation of the irritant receptor TRPA1. Proc Natl Acad Sci USA.

[B31] Gajraj NM (2007). Pregabalin: its pharmacology and use in pain management. Anesth Analg.

[B32] Kim SH, Chung JM (1992). An experimental model for peripheral neuropathy produced by segmental spinal nerve ligation in the rat. Pain.

[B33] Chaplan SR, Bach FW, Pogrel JW, Chung JM, Yaksh TL (1994). Quantitative assessment of tactile allodynia in the rat paw. J Neurosci Methods.

