# Supplementary material for: HC-030031, a TRPA1 selective antagonist, attenuates inflammatory- and neuropathy-induced mechanical hypersensitivity
Source: Mol Pain. 2008 Oct 27;4:48. doi: 10.1186/1744-8069-4-48 (PMC2584039; doi:10.1186/1744-8069-4-48)
Supplement: Additional file 1 — Table 1: In-vitro pharmacological selectivity of HC-030031. Radioligand binding or enzymatic assays results are summarized as the percent inhibition of specific binding or enzymatic activity. HC-030031 exhibited no significant activity in all assays employed (significance criteria is ≥ 50% of maximal stimulation or inhibition; MDS Pharma Service, Taipei, Taiwan.). [file 1744-8069-4-48-S1.pdf]

Additional Table 1.

Target name	Species	Tissues	Concentration	% inhibition	Assay
Cyclooxygenase COX-2	Human	recombinant	10 μM	-16	Enzymatic
Fatty Acid Amide Hydrolase (FAAH)	Rat	brain	10 μM	-5	Enzymatic
Nitric Oxide Synthase, Neuronal (nNOS)	Rat	cerebellum	10 μM	23	Enzymatic
Phospholipase PLA2-II	Crotalus atrox		10 μM	-4	Enzymatic
Protein Serine/Threonine Kinase, MAPK1 (ERK2)	Human	recombinant	10 μM	5	Enzymatic
Protein Serine/Threonine Kinase, IKK-2	Human	recombinant	10 μM	23	Enzymatic
Protein Serine/Threonine Kinase, MAPK11 (p38beta)	Human	recombinant	10 μM	1	Enzymatic
Adenosine A1	Human	recombinant	10 μM	-1	Radioligand Binding
Adrenergic alpha1, Non-Selective	Rat	brain	10 μM	2	Radioligand Binding
Adrenergic alpha2, Non-Selective	Rat	cerebral cortex	10 μM	12	Radioligand Binding
Bradykinin B1	Human	IMR-90 cells	10 μM	4	Radioligand Binding
Bradykinin B2	Human	recombinant	10 μM	7	Radioligand Binding
Calcitonin Gene-Related Peptide CGRP1	Human	SK-N-MC cells	10 μM	-10	Radioligand Binding

Calcium Channel L-Type, Dihydropyridine	Rat	cerebral cortex	10 μM	1	Radioligand Binding
Calcium Channel N-Type	Rat	brain frontal lobe	10 μM	2	Radioligand Binding
Cannabinoid CB1	Human	recombinant	10 μM	0	Radioligand Binding
Cannabinoid CB2	Human	recombinant	10 μM	33	Radioligand Binding
Chemokine CCR1	Human	recombinant	10 μM	7	Radioligand Binding
Chemokine CCR2B	Human	recombinant	10 μM	-8	Radioligand Binding
Chemokine CCR5	Human	recombinant	10 μM	11	Radioligand Binding
Cholecystokinin CCK2 (CCKB)	Human	FGS-7 Jurkat cells	10 μM	-2	Radioligand Binding
Dopamine D1	Human	recombinant	10 μM	-2	Radioligand Binding
Dopamine D2L	Human	recombinant	10 μM	5	Radioligand Binding
GABAA, Muscimol, Central	Rat	brain (minus cerebellum)	10 μM	-1	Radioligand Binding
GABAB, Non-Selective	Rat	brain	10 μM	3	Radioligand Binding
Gabapentin	Rat	brain cortex	10 μM	-1	Radioligand Binding
Glutamate, Non-Selective	Rat	brain	10 μM	7	Radioligand Binding
Glycine, Strychnine-Sensitive	Rat	spinal cord	10 μM	25	Radioligand Binding
Interleukin IL-1	Mouse	3T3 cells	10 μM	6	Radioligand Binding
Interleukin IL-6	Human	U266 cells	10 μM	1	Radioligand Binding
Transporter, Monoamine	Rabbit	platelets	10 μM	37	Radioligand Binding
Muscarinic, Non-Selective, Central	Rat	cerebral cortex	10 μM	2	Radioligand Binding
Tachykinin NK3	Human	recombinant	10 μM	-2	Radioligand Binding
Nicotinic Acetylcholine	Human	IMR-32 cells	10 μM	-6	Radioligand Binding
Opiate, Non-Selective	Rat	brain	10 μM	21	Radioligand Binding
Orphanin ORL1	Human	recombinant	10 μM	-10	Radioligand Binding
Prostanoid EP2	Human	recombinant	10 μM	-4	Radioligand Binding

Prostanoid EP4	Human	recombinant	10 µM	4	Radioligand Binding
Purinergic P2X	Rabbit	urinary bladder	10 µM	7	Radioligand Binding
Purinergic P2Y	Rat	brain	10 µM	6	Radioligand Binding
Serotonin (5-Hydroxytryptamine) 5-HT1, Non-Selective	Rat	cerebral cortex	10 µM	-1	Radioligand Binding
Serotonin (5-Hydroxytryptamine) 5-HT2, Non-Selective	Rat	brain	10 µM	-1	Radioligand Binding
Serotonin (5-Hydroxytryptamine) 5-HT3	Human	recombinant	10 µM	-12	Radioligand Binding
Sigma, Non-Selective	Guinea pig	brain	10 µM	37	Radioligand Binding
Sodium Channel, Site 2	Rat	brain	10 µM	40	Radioligand Binding
Tumor Necrosis Factor (TNF), Non-Selective	Human	U937 cells	10 µM	-8	Radioligand Binding
Vanilloid	Rat	spinal cord	10 µM	-2	Radioligand Binding
Vasoactive Intestinal Peptide VIP1	Human	HT29 colon carcinoma cells	10 µM	4	Radioligand Binding
